# Monitoring Pancreatic Carcinogenesis by the Molecular Imaging of Cathepsin E *In Vivo* Using Confocal Laser Endomicroscopy

**DOI:** 10.1371/journal.pone.0106566

**Published:** 2014-09-03

**Authors:** Hui Li, Yongdong Li, Lei Cui, Biyuan Wang, Wenli Cui, Minghua Li, Yingsheng Cheng

**Affiliations:** 1 Department of Radiology, the Sixth Affiliated People's Hospital, Shanghai Jiao Tong University, Shanghai, China; 2 College of Science, University of Shanghai for Science and Technology, Shanghai, China; 3 Department of Pathology, Fudan University Shanghai Cancer Center, Shanghai, China; Stanford University, United States of America

## Abstract

The monitoring of pancreatic ductal adenocarcinoma (PDAC) in high-risk populations is essential. Cathepsin E (CTSE) is specifically and highly expressed in PDAC and pancreatic intraepithelial neoplasias (PanINs), and its expression gradually increases along with disease progression. In this study, we first established an *in situ* 7,12-dimethyl-1,2-benzanthracene (DMBA)-induced rat model for PanINs and PDAC and then confirmed that tumorigenesis properties in this model were consistent with those of human PDAC in that CTSE expression gradually increased with tumor development using histology and immunohistochemistry. Then, using *in vivo* imaging of heterotopically implanted tumors generated from CTSE- overexpressing cells (PANC-1-CTSE) in nude mice and *in vitro* imaging of PanINs and PDAC in DMBA-induced rats, the specificity of the synthesized CTSE-activatable probe was verified. Quantitative determination identified that the fluorescence signal ratio of pancreatic tumor to normal pancreas gradually increased in association with progressive pathological grades, with the exception of no significant difference between PanIN-II and PanIN-III grades. Finally, we monitored pancreatic carcinogenesis *in vivo* using confocal laser endomicroscopy (CLE) in combination with the CTSE-activatable probe. A prospective double-blind control study was performed to evaluate the accuracy of this method in diagnosing PDAC and PanINs of all grades (>82.7%). This allowed us to establish effective diagnostic criteria for CLE in PDAC and PanINs to facilitate the monitoring of PDAC in high-risk populations.

## Introduction

The onset of pancreatic ductal adenocarcinoma (PDAC) usually goes undetected. As such, the majority of patients that present with advanced-stage disease have a poor prognosis. Invasive PDAC is nearly always a fatal condition, and although it comprises only 3% of estimated new cancer cases each year, it is the fourth most common cause of cancer mortality in the United States [1].

Because of its relatively low incidence, current research is focused on the early detection and screening of patients at high risk of developing PDAC [2]. Risk factors for PDAC include a family history of PDAC; >40 years of age with nonspecific abdominal discomfort; sudden diabetes, especially atypical forms; chronic pancreatitis, especially hereditary chronic pancreatitis and chronic calcifying pancreatitis; patients with familial adenomatous polyposis; long-term smoking; heavy alcohol intake; and long-term exposure to hazardous chemicals [3]–[5].

More than 95% of cases of pancreatic adenocarcinoma are PDAC that originates from pancreatic ductal epithelium, which develops in a multi-stage process. Pancreatic intraepithelial neoplasia (PanIN) is the main precancerous lesion of PDAC, which further develops into early PDAC. These lesions are divided into low-grade PanIN (PanIN-I) and high-grade PanIN (PanIN-II and PanIN–III), according to the progression of pathomorphological changes [6], [7].

Clinical research studies [8], [9] have found a high discovery rate of PanINs in pancreatic tissue removed from patients at a high risk of developing PDAC who undergo the partial or total removal of the pancreas for an unrelated reason. In addition, for those patients without invasive PDAC, surgery to remove PanINs has a high cure rate. Therefore, the monitoring of PDAC in high-risk populations is essential to improve the prevention and cure of this condition. However, despite advances in screening and the early detection of other cancers, such as breast and colon cancer, no reliable screening test exists for PDAC at present.

Cathepsin E (CTSE) is highly and specifically expressed in PDAC and PanINs, and its expression gradually increases with disease progression [10]. Various CTSE- optical molecular probes have been successively developed in recent years. Tung et al [10], [11]. constructed a smart optical molecular probe for the specific detection of CTSE. Under the catalysis of CTSE, their probe peptide (a distinct CTSE-selecting substrate) bonds were hydrolyzed to release fluorophores, which release a fluorescent signal. They then succeeded in detecting early PDAC and PanINs in transgenic mice by *in vitro* imaging for the probe signal [12], [13].

The United States Food and Drug Administration (FDA)-approved human immunodeficiency virus (HIV) protease inhibitor ritonavir (RIT) has a high affinity for CTSE. Therefore, Weissleder et al. have developed a CTSE optical imaging agent, ritonavir tetramethyl-BODIPY (RIT-TMB), which can identify the tumor margin to assess the degree of infiltration during surgery [14].

In this research, we initially synthetized this specific CTSE-activatable probe according to the method described by Tung et al [10]. We then conducted *in vivo* imaging of heterotopically implanted tumors generated from the cells that overexpress CTSE (PANC-1-CTSE) in nude mice. Fluorescence signal changes were detected *in vitro* in 7,12-dimethyl-1,2-benzanthracene (DMBA)-induced rat PanINs of various stages and early PDAC. We further pioneered the use of *in vivo* imaging techniques in combination with confocal laser endomicroscopy (CLE) for the continuous monitoring of pancreatic carcinogenesis in rats.

## Material and Methods

### Ethics statement

Animal procedures were performed in accordance with the recommendations in the Guide for the Care and Use of Laboratory Animals published by the National Institutes of Health. The animal study was approved by the Institutional Animal Care and Use Committees of the China Medical Association (Permit Number: CCMA 2012–2397).

### Preparation of CTSE-fluorescent probes

All solvents used were of analytical or HPLC grade. Dichloromethane (DCM), N-methylpyrrolidone (NMP) and methanol (MeOH) were purchased from Sinoreagent (Shanghai, China). N, N-Dimethylformamide (DMF), diethylether, acetonitrile (MeCN), diisopropylethylamine (DIPEA), DL-dithiothreitol (DTT), triethylacetate (TEA), dimethylsulfoxide (DMSO), iodoacetic anhydride, piperidine Triisopropylsilane (TIS), triethylamine (TEA) and 1,2-ethanedithiol (EDT) were purchased from Sigma-Aldrich (Shanghai, China). HOBt and HBTU were purchased from Energy Chemical (Shanghai, China). Fmoc-protected amino acids were custom synthesized by GL Biochem (Shanghai, China). D-Polylysine was purchased from Sigma-Aldrich (Shanghai, China).

**Figure 1:**
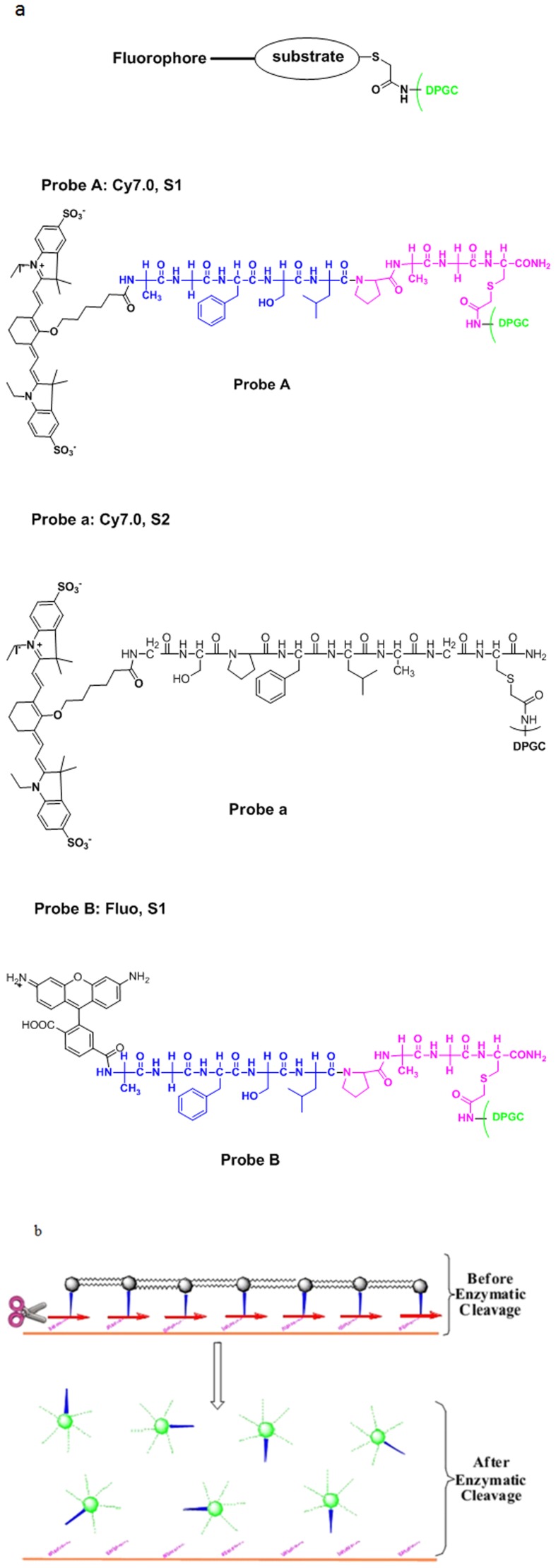
The probe chemical structure and action mechanism. (a) Chemical structures of optical probes. (b) Action mechanism of CTSE optical probe.

**Figure 2:**
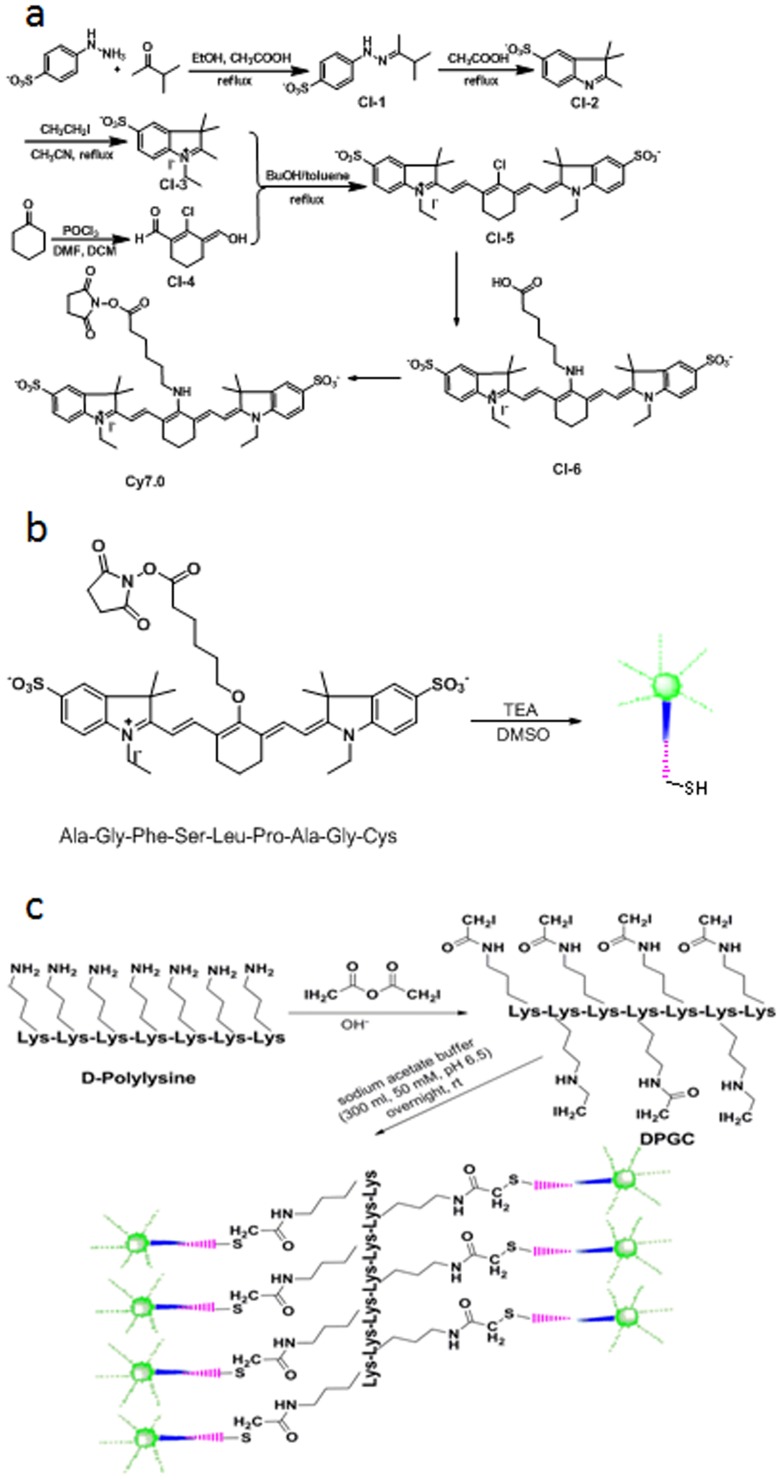
Concrete synthesis procedures of CTSE optical imaging probe A. (a) Synthesis of Cy7.0. (b) Synthesis of Cy7.0-labelled peptide substrate. (c) Surface modification of poly-lysine and conjugation with Cy7.0-labelled peptide substrate.

In order to meet the needs of two different instruments for tests, Cy7.0 (probe A and probe a) and 6-carboxyl fluorescein succinimide ester (probe B) were selected as fluorophores, with excitation wavelengths of near-infrared and 490 nm, respectively. Peptide substrates, Ala-Gly-Phe-Ser-Leu-Pro-Ala-Gly-Cys-CONH2 (probe A, a selective peptide substrate for CTSE), Gly-Ser-Pro-Phe-Leu-Ala-Gly-Cys-CONH2 (probe a, a selective peptide substrate for cathepsin D), were synthesized by solid-phase peptide synthesis (SPPS) on Rink amide Novagel resin (0.1 mmole, 0.61 mmol mg_1) using standard Fmoc chemistry with HBTU/HOBT coupling on an automatic synthesizer (ABI-433A, Applied Biosystems). The peptides were purified by HPLC and then Cy7.0-NHS ester was coupled to the N-termini in anhydrous DMSO and TEA overnight in the dark. After HPLC purification, the identity of Cy7.0 labeled peptides was confirmed by LC-MS using a Thermo Finnigan LCQ Fleet mass spectrometer (Thermo-Fisher Scientific, West Palm Beach, FL, USA). The results revealed the high purity (>94%) and confirmed the identities of the precursor peptides. The probe preparation protocol is similar to the previously reported protocol for other protease sensitive probes [15]. Briefly, iodoacetylated- D-Polylysine (DPGC) was prepared by reacting iodoacetic anhydride with D-Polylysine (1 mg, MW = 60 KDa) under basic conditions, and then the prepared DPGC was reacted with Cy7.0-peptide substrates (1 mg) in sodium acetate buffer (300 ml, 50 mM, pH 6.5) overnight at room temperature in the dark. The formed imaging probes were collected by membrane cutoff filtration and quantified by measuring Cy7.0 molar absorbance at 762 nm. Peptide loadings on DPGC were calculated using the relative mole ratio of fluorochrome to D-Polylysine. On average, 21–23 peptides were attached to each D-Polylysine molecule. The quenching efficiency of the probes was quantified by measuring the fluorescence signal intensity of imaging probes to that of free equal-moles of Cy7.0 dye. More than 95% of fluorescent quenching was obtained. For probe B, the 6-carboxyl fluorescein succinimide ester was used as the fluorophore polymer, the peptide was similar as probe A, and was synthesized as described above. (Fig 1, 2, Table 1)

**Table 1:** Peptide substrates used for imaging probes preparation and their characterization.

Probe	Protease-selective peptide sequence	Calculated mol. wt.	Observed [M+H]^+^or [M]^+^
A	Cy7.0-Ala-Gly-Phe-Ser-Leu-Pro-Ala-Gly-Cys-CONH_2_	1567.67	1567.65
a	Cy7.0-Gly-Ser-Pro-Phe-Leu-Ala-Gly-Cys-CONH_2_	1481.62	1481.66
B	Fluo-Ala-Gly-Phe-Ser-Leu-Pro-Ala-Gly-Cys-CONH_2_	1177.48	1178.52

### Generation of cell line that overexpresses CTSE (PANC-1-CTSE), Western blot studies and heterotopic implantation

Human CTSE (NM_001910.3) was synthesized (Life Technologies) and cloned into a *Nhe*I/*Asc*I-digested pLenti6.3_MCS_IRES2-EGFP plasmid using the In Fusion Cloning Kit (Clontech, Mountain View, CA). Ligated DNA was transformed into DH5α cells using the rapid transformation protocol, as recommended by the manufacturer (Life Technologies). Plasmid DNA was extracted from ampicillin-resistant colonies grown first on Luria-Bertani agar plates and then in Luria-Bertani broth, and plasmids were verified by DNA sequencing (Life Technologies).

Lentiviral particles were generated by transfecting the HEK293T cell line with packaging vectors (pLP1, pLP2, pLP/VSVG; obtained from Life Technologies) and pLenti6.3_CHSE_IRES2-EGFP for approximately 3 days, at which point samples of media were collected and filtered through a 0.45-µm cellulose acetate syringe filter, aliquoted into cryotubes, and snap frozen in liquid nitrogen.

PANC-1 human pancreatic adenocarcinoma cells (1×10^4^ cells/well, purchased from the Chinese Academy of Sciences cell bank) with a low expression level of CTSE were seeded in a 12-well plate and transduced with lentivirus in a 1∶1 ratio with growth medium with 8 µg/ml polybrene. After 72 hours, PANC-1-CTSE cells were generated, and then trypsinized and split into dishes with growth medium (DMEM medium with 10% FBS and 1% glutamine/penicillin/streptomycin).

Cultured PANC-1 cells were dissociated into single cells with trypsin-EDTA and analyzed under fluorescence microscopy to evaluate transduction efficiency. The conditions for lentiviral transduction were as follows; PANC-1 was infected with lentivirus with seven different MOIs (5, 10, 20, 50, 100, 200 or 300) for 72 h. After determining the optimal condition for infection (MOI = 50), we examined the effects of the feeder layer on the proficiency of transduction. The transduction efficiency was evaluated based on the number of GFP-expressing PANC-1 cells as scored under fluorescence microscopy.

For whole-cell protein extraction, cells were washed with cold PBS and subsequently lysed in cold RIPA lysis buffer (50 mM Tris–HCl, pH 7.4, 150 mM NaCl, 1 mM dithiothreitol [DTT], 0.25% sodium deoxycholate and 0.1% NP-40) containing 1 mM phenylmethysulfonyl fluoride (PMSF), 50 mM sodiumpyrophosphate, 1 mM Na3VO4, 1 mM NaF, 5 mM EDTA, 5 mM EGTA, and a protease inhibitor cocktail (Roche Molecular Biochemicals, Mannheim, Germany). Cell lysis was performed on ice for 30 minutes. Clear protein extracts were obtained by centrifugation for 30 minutes at 4°C. Protein concentrations were determined by the Bradford method using the Bio-Rad protein assay reagent (Bio-Rad Laboratories, Hercules, CA, USA) and 20–40 mg of protein mixed with loading buffer was loaded per lane, separated by 12% SDS-polyacrylamide gel electrophoresis (SDS-PAGE). Proteins were transferred to PVDF membrane filters (Millipore, USA). Nonspecific binding was blocked by incubation in phosphate-buffered saline (PBS) containing 0.1% Tween 20 (PBS-T) and 5% skim milk. PVDF membranes were blocked with 5% dry milk for 1 hour at 4°C. Membranes were incubated in anti-cathepsin E primary antibody (Cath E antibody [1∶100 dilution; sc-166500; Santa Cruz Biotechnology, Santa Cruz, California, USA]) overnight at 4°C. The membranes were then incubated with the corresponding secondary antibody (1∶2000, anti-GAPDH-HRP) in TBST-5% non-fat milk for 1 hour at room temperature and the immunoreactive bands were visualized using EZ ECL Chemiluminescence Detection Kit for HRP (Thermo, US). Images were acquired using the Kodak X-OMAT BT (Kodak). Membranes were re-probed using β-actin as a loading control.

PANC-1 cells and PANC-1-CTSE cells were cultured in growth medium, counted with a cytometer, and the cell concentration was adjusted to 1×10^6^ cells/50 µl. PANC-1-CTSE cells and PANC-1 cells were subcutaneously injected into twenty nude mice (male BALB/C-nu/nu mice, 6 weeks of age, weighing 19–22 g) at the right thigh root and the right axilla, respectively, for heterotopic implantation. The mice were inspected daily until tumors were grown to 3–5 mm, as measured by calipers.

### Assessment of CTSE-fluorescent probes

The 20 mice were randomly devided into four groups. Probe a (4 mg/kg, group 1), probe A (20 mg/kg, group 2), probe A (4 mg/kg, group 3), and probe A (2 mg/kg, group 4) were respectively injected into the mice via the tail vein. After 72 h, mice were injected intraperitoneally with a mixture of ketamine (100 mg/kg) and xylazine (10 mg/kg) for deep anesthesia. *In vivo* NIR imaging (Kodak-*In Vivo* Multispectral Imaging System, Carestream Health Inc., USA) and quantitative determination (software: Carestream MI) were then conducted.

### Preparation and pathological confirmation of *in situ* DMBA-induced PDAC in rats

Rats (SD, male, weighing 200 g, n = 75) were intraperitoneally injected with a mixture of ketamine (100 mg/kg) and xylazine (10 mg/kg) to induce a deep anesthesia. The epigastrium was disinfected and a median incision was made at the abdomen to expose the pancreas. The pancreatic capsule at the tail of the pancreas was carefully cut, and a DMBA particle (Sigma Pharmaceuticals, South Croydon, Victoria, Australia) at a dose of 5 mg/100 g was placed in it. A purse string suture was performed around the incision, which was then tightened and ligated, before the abdominal cavity was closed. For the control group (n = 5), the purse string suture was carried out without the DMBA implant. The rats were sacrificed with excess ketamine-xylazine at 1, 2, 3, 4 and 5 months after surgery (n = 15 for the experimental group, and n = 1 for the control group for each time point). The tissue surrounding the purse string suture was sampled, then used for formalin fixation and paraffin embedding, followed by HE staining, histopathological examination, CTSE immunohistochemical analysis and Western blot studies.

### CTSE immunohistochemical analysis and Western blot studies

FFPE (formalin-fixed and paraffin-embedded) samples were collected onto poly-L-lysine coated slides and after a heat-induced antigen retrieval procedure, with EDTA for pAKT, and citric acid for other antigens, for 3 min, were processed with a standard manual streptavidin-peroxidase technique using a biotin-free detection system (Dako, Carpinteria, CA, USA). Samples were incubated with primary antibodies at 4°C overnight. For a negative control, the primary antibody was replaced with an antibody diluent. A ready-to-use kit (EnVisionTM; Dako) was used in combination with the primary antibody (Cath E antibody [1∶200 dilution; sc-166500; Santa Cruz Biotechnology, Santa Cruz, California, USA]), according to the manufacturer's instructions.

Proteins were isolated from five tissue types (normal pancreas, PanIN-I, PanIN-II, PanIN–III, and PDAC) using the Tissue Protein Extraction Kit (3W0819A, CWBIO, China) according to the manufacturer's instruction following by Western blotting assays as described above.

### *In vitro* imaging and pathological comparison of tumorigenesis of DMBA-induced PDAC in rats injected with probe A and probe a

During pancreatic carcinogenesis in the DMBA-induced rats (n = 30), *in vitro* imaging was carried out at 1, 2, 3, 4, and 5 months after DMBA placement, as well as at various stages of pancreatic carcinogenesis. The molecular probes (probe A and probe a, 2 mg/ml in PBS) were randomly injected into the tail vein of rats (n = 4×5 for probe A, and n = 2×5 for probe a). After 72 hours, the animals were sacrificed via excess ketamine-xylazine, and samples of the tumor tissue, liver, kidney, and muscle were collected for *in vitro* imaging and quantitative determination. Finally, HE staining, histopathological examination and CTSE immunohistochemical analysis were performed.

### *In vivo* imaging of rats with DMBA-induced PDAC using CLE

The handheld micro CLE probe was placed on the surface of the pancreatic lesions for single-cell-resolution imaging, combined with the specific CTSE-activatable probe. The CLE images were then analyzed for intensity of the fluorescence signal, morphology and size of the CTSE-positive cells, and fluorescent activation patterns (whether homogeneous or not).

1. Dynamic *in vivo* imaging with CLE

During pancreatic carcinogenesis in the DMBA-induced rat model (n = 5), dynamic *in vivo* imaging with CLE (Cellvizio Image Cell, Mauna Kea Technology Inc., France) was carried out at various stages of pancreatic carcinogenesis (day 0, 30 days, 60 days, 90 days, 120 days, or 150 days after DMBA placement). The molecular probe (probe B, 2 mg/ml in PBS) was intravenously injected, then after 72 hours, rats were fixed after inducing a deep anesthesia; the left upper abdominal wall was disinfected, a small incision was made, and the handheld micro CLE probe, which scans and records the signals on the surface of and inside the tumor layer-by-layer, was inserted. The scanning parameters were an excitation wavelength of 488 nm, and acquisition bandwidth of 505–585 nm. Finally, the abdomen was closed, animals continued feeding and observed.

1. Prospective, double-blind control study using CLE

DMBA was induced at day 0, 30, 60, 90, and 120 in rats (n = 8×5 for the experimental group; n = 2×5 for the control group), and at day 150, all animals (n = 47; there were 3 deaths after day 100) were killed with excess anesthesia after *in vivo* CLE imaging (i.e. imaging at the first, second, third, fourth, fifth month after induction). Samples of PDAC tissue were collected for HE staining, histopathological examination and CTSE immunohistochemical analysis. CLE researchers (HL and BW) and pathology researcher (WLC) who evaluated the tumor grade based on CLE (blinded to the pathological results) and pathological findings (blinded to the CLE results), respectively, were not aware of the induction time.

Image J was used for the quantitative analysis of the fluorescence intensity of CLE images. For images of every observed lesion and its surrounding normal pancreatic tissue, researchers selected images with minimal motion artifact and strong fluorescent signal for analysis. The average fluorescence intensity was calculated.

### Statistical analysis

SPSS statistical software (version 13.0) was used for the statistical analysis of data. Statistically significant differences were determined by two-tailed unpaired Student's t test; the level of significance was set at *p*<0.05. Cohen's kappa value was calculated to assess the reliability of data collected between the two observers.

## Results

### Assessment of the validity and specificity of the CTSE-activatable probe using the heterotopic implantation of PANC-1-CTSE cells into nude mice

**Figure 3:**
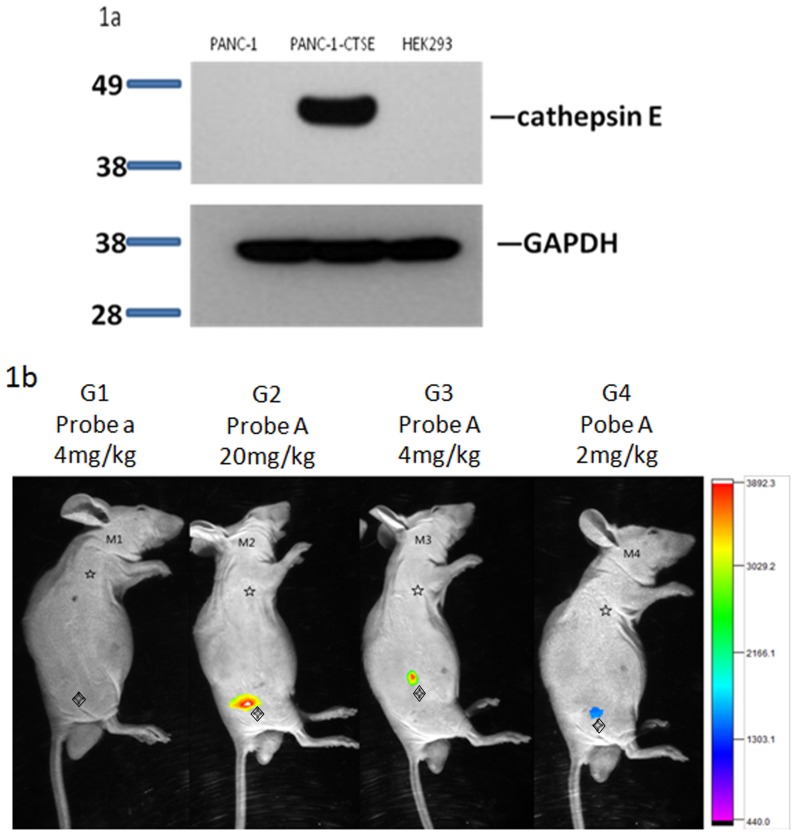
Near-infrared in vivo fluorescence imaging of heterotopically transplanted pancreatic adenocarcinoma in nude mice. (a) Western blot shows the overexpression of CTSE in PANC-1-CTSE cells, which was much higher than that in PANC-1 and HEK293 cells (negative). (b) Four groups of nude mice (G1-G4) were all subcutaneously injected with PANC-1-CTSE and PANC-1 cell suspensions in the right thigh root (◊) and right axilla (☆), respectively. One group of nude mice (G1) was injected with probe a (4 mg/kg), while the others were injected with different doses of the probe A as follows: G2: 20 mg/kg; G3: 4 mg/kg; G4: 2 mg/kg. Abnormally high signals were observed in the right thigh root of the mice injected with probe A (G2-M4), with similar values observed in the G2 and G3. These were both significantly higher than that observed in G4. The signals were absent in the right thigh root of the mice injected with probe a (G1) and in the right axilla of all mice (G1–G4).

PANC-1-CTSE cells demonstrated immunoreactive bands at 43 KDa, which was the anticipated molecular weight of CTSE. Densitometry studies showed that the expression levels of CTSE in PANC-1-CTSE cells were much higher than in PANC-1 cells (Fig. 3a).

In the nude mice injected with probe A, the probe was accumulated in the PANC-1-CTSE tumors, whereas the control PANC-1 tumors showed no probe accumulation. In the nude mice injected with probe a, both tumor types showed no probe accumulation (Fig. 3b).

In addition, similar levels of probe accumulation were observed in the PANC-1-CTSE tumors in mice injected with 20 mg/kg (signal level: 327±23) and 4 mg/kg (signal level: 295±18) of probe A, and both levels were significantly higher (*p* = 0.005, 0.012) than those observed in the mice injected with 2 mg/kg (signal level: 116±15) of probe A (Fig. 3b). Therefore, we determined that the optimal injection dose for the probes was 8 mg/kg.

### Histopathological, immunohistochemical analysis and Western blot studies for the assessment of tumorigenesis and changes of CTSE expression during *in situ* DMBA-induced PDAC in rats

**Figure 4:**
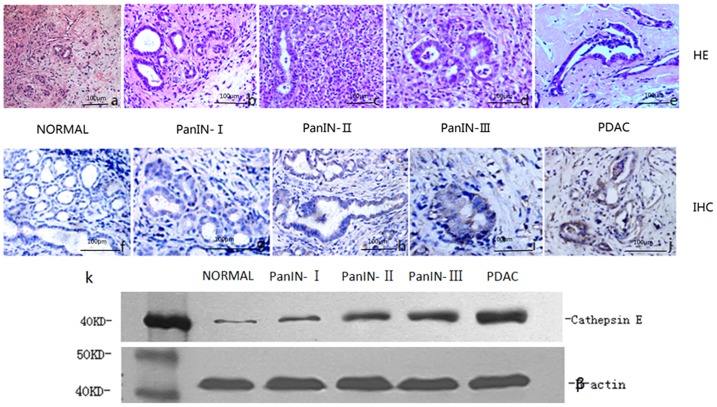
HE staining, immunohistochemical analysis and Western blot studies of CTSE of *in situ* DMBA-induced PDAC and PanINs. (a-e) Representative images of histology (HE staining) in pancreatic tissue sections of (a) normal pancreas; (b) PanIN-I; (c) PanIN-II; (d) PanIN-III; and (e) PDAC. (f-j) Representative images of immunohistochemical localization of CTSE in pancreas tissue sections of (f) normal pancreas; (g) PanIN-I; (h) PanIN-II; (i) PanIN-III; and (j) PDAC, showing a gradually increasing trend of CTSE expression (brown) with increasing grade of maglignancy. (k) Western blot shows very low levels in normal pancreatic tissues, but higher levels in PanINs and PDAC tumors. Scale bar, 100 µm.

In this experiment, histopathological examination of pancreatic tissue was carried out after the *in situ* induction of DMBA. A large number of infiltrating inflammatory cells and interstitial connective tissue hyperplasia were visible in local pancreatic tissue; and there was evidence of the pathological changes of chronic pancreatitis, consistent with the histological features of human PDAC. After the establishment of the tumor model, a proportion of rats developed PanIns at 1–2 months, and PDAC at 3–4 months (Table 2 and Figs. 4a-e).

**Table 2:** Process of *in situ* DMBA-induced pancreatic adenocarcinoma in rats.

	Normal	PanIN-I	PanIN-II	PanIN-III	PDAC		Other
					Infiltration	Distant metastasis	
30^th^ day	2/15	9/15	4/15	0/15	0/15	0/15	-
60^th^ day	0/15	1/15	2/15	11/15	1/15	0/15	-
90^th^ day	0/14	0/14	3/14	4/14	6/14	1/14	One death
120^th^ day	0/9	0/9	1/9	2/9	2/9	4/9	Six death
150^th^ day	0/6	0/6	0/6	0/6	0/6	6/6	Nine death

Abbreviations: DMBA, 7,12-dimethyl-1,2-benzanthracene; PanIN, pancreatic intraepithelial neoplasia; PDAC, pancreatic ductal adenocarcinoma.

Immunohistochemical analysis and Western blot studies were then performed to measure the expression of CTSE (Figs. 2f-k). The results showed that CTSE expression was very low in normal pancreatic tissue, but high in PanINs and PDAC tumors, where CTSE expression level showed an increasing trend along with the progressive grade.

### Assessment of the validity and specificity of the CTSE-activatable probe using the DMBA-induced PDAC models

**Figure 5:**
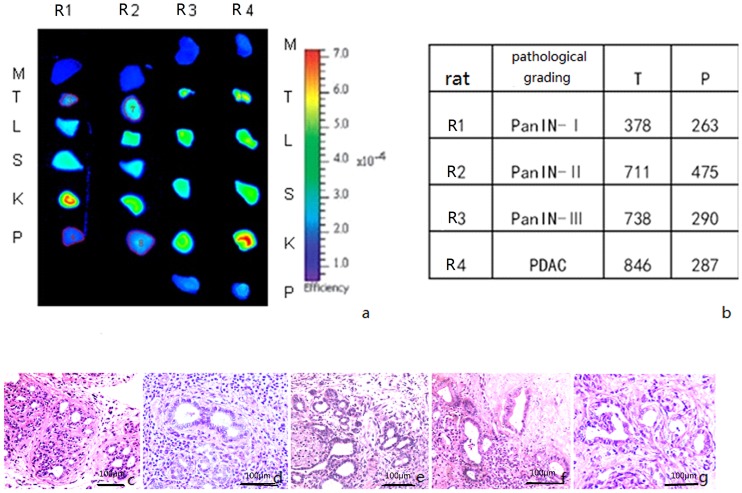
*In vitro* imaging and quantitative determination of PDAC and PanINs in DMBA-induced rats injected with probe A. (a) *In vitro* imaging of tissue from tumors (R1 = PanIN-I; R2 = PanIN-II; R3 = PanIN-III; R4 = PDAC), kidney, liver, spleen, muscle and normal pancreas of four rats. (b) Quantitative analysis of near-infrared fluorescence imaging of tumor tissue and normal pancreatic tissue from four rats. (c-g) Routine pathological HE staining of (c) normal pancreas; (d) PanIN-I (R1); (e) PanIN-II (R2); (f) PanIN-III (R3); and (g) PDAC (R4). M = muscle; T = tumor; L = liver; S = spleen; K = kidney; P = pancreas. Scale bar, 100 µm.

**Figure 6:**
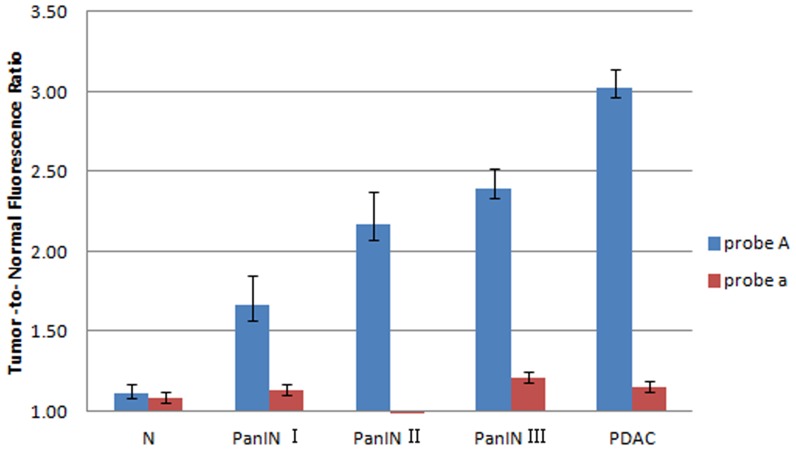
Quantitative determination curve of the *in vitro* imaging fluorescence signal of DMBA-induced PDAC and PanINs in rats injected with probe A and probe a. The results showed that the fluorescent signal ratio of tumor:normal pancreatic parenchyma increased with progressive pathological grade in rats injected with probe A. On the other hand, the ratio was almost unchanged in rats injected with probe a. Error bars represent SD. N = normal.

For the near-infrared *in vitro* imaging, fluorescence signals were descending in the following normal organs: kidney, liver, spleen, normal pancreas, and muscle. In rats injected with probe A, the fluorescence signals of the pancreatic tumors showed an increasing trend as the degree of their malignancy increased (Fig. 5). Through quantitative determination, the ratio of pancreatic tumor:normal pancreas gradually increased in association with progressive tumor grade (Fig. 4), except for no significant difference between the PanIN-II and PanIN-III grades (*p* = 0.076). In brief, the low- and high-grade PanINs and PDAC could be identified by *in vitro* fluorescent imaging using probe A. Comparatively, in rats injected with probe a, the fluorescence signals of the pancreatic tumors were uniformly faint throughout, and most were slightly higher than the signals of normal pancreatic parenchyma (Fig. 6).

### Dynamic observation of pancreatic carcinogenesis through *in vivo* imaging of *in situ* DMBA-induced PDAC in rats using CLE with the CTSE probe (probe B)

CLE continuous dynamic *in vivo* imaging was used to monitor the development of DMBA-induced PDAC in rats. We found that CTSE-positive cells were gradually increased with the delay of induction time, and the fluorescence signal intensity was increased and uneven (Videos S1–S6).

### Assessing the diagnostic accuracy of CLE combined with CTSE molecular probe (probe B) on DMBA-induced PDAC in rats

**Figure 7:**
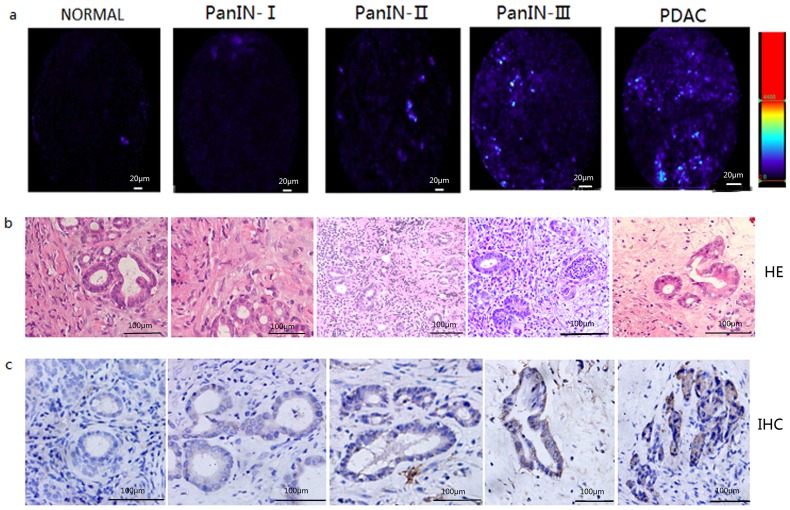
*In vivo* CLE imaging of DMBA-induced PDAC in rats and pathological comparison. (a) *In vivo* CLE imaging of normal pancreas, low- and high-grade PanIN lesions, and PDAC (blue) with the activated CTSE-sensitive probe. (b) Representative images of histology in pancreas tissue sections from normal pancreas, low- and high-grade PanIN lesions, and PDAC. (c) Representative images of immunohistochemical localization of CSTE in pancreas tissue sections from normal pancreas, low- and high-grade PanIN lesions, and PDAC. Scale bar, 100 µm.

The pathological data suggested (based on the highest pathological grade of lumps): of the 40 rats in the experimental group, except for the four deaths (one rat died at the 82^th^ day after transplant, one at the 111^th^ day, and two at the 130^th^ day), a total of three were normal, there were nine low-grade cases (PanIn-I), sixteen high-grade cases (including PanIn-II and PanIn-III) and eight of PDAC; while for ten rats in the control group, a total of nine were normal and there was one low grade case (PanIn-I) (Fig. 7 and Table 3).

**Table 3:** Comparison between pathological and CLE results.

	Normal	Low-grade PanIN	High-grade PanIN	PDAC
Pathology	12	10	16	8
CLE-1	11+1(low) ^*^^*^	1(N) ^*^+8+1(high) ^*^^*^	1(low) ^*^+14+1(cancer) ^*^^*^	8
CLE-2	12	9+1(high) ^*^^*^	1(low) ^*^+14+1(cancer) ^*^^*^	1(high) ^*^+7

The results for CLE-1 and CLE-2 were the assessments of two researchers, respectively. Mistaken assessments are shown in brackets; ^*^ false negative; ^*^^*^ false positive.

Abbreviations: CLE, confocal laser endomicroscopy; PanIN, pancreatic intraepithelial neoplasia; PDAC, pancreatic ductal adenocarcinoma.

**Figure 8:**
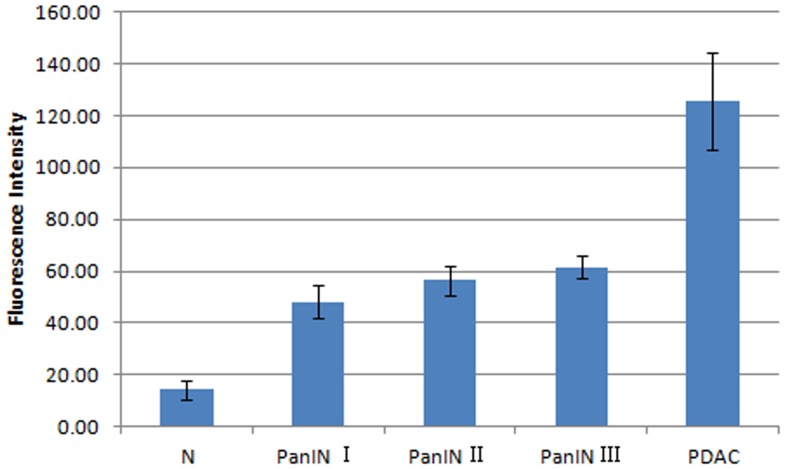
Average fluorescence intensity of *in vivo* CLE imaging of DMBA-induced rat PDAC and PanINs. The average fluorescence intensity gradually increased with the pathological grade with the exception of no significant difference between PanIN-II and PanIN-III grades (*p* = 0.140). Error bars represent SD. N  =  normal.

The accuracy of the two researchers assessing PanINs grading was higher than 82.7% (Table 4), and the consistency between the two observers was 73.8%. We made a retrospective quantitative analysis of the fluorescent signals of the lesions. The average fluorescence intensity was 48.0 for PanIN-I; 56.2 for PanIN-II; 61.5 for PanIN-III; and 125.7 for PDAC. In contrast, the fluorescence intensity of normal pancreatic tissue surrounding the lesions was 14.2. The difference in fluorescence intensity between PanIN-II and PanIN-III was not significant (*p* = 0.140) (Fig. 8).

**Table 4:** Assessment of two researchers on the CLE images, including the average sensitivity, specificity, positive predictive value, negative predictive value and accuracy.

	Sensitivity	Specificity	PPV	NPV	Accuracy
Normal	92.1	94.6	92.4	94.5	92.2
Low-grade	90.3	92.1	82.6	95.5	82.7
High-grade	88.2	88.7	82.9	92.3	82.7
Cancer	87.5	92.1	91.7	95.3	91.2

Abbreviations: CLE, confocal laser endomicroscopy; PPV, positive predictive value; NPV, negative predictive value.

## Discussion

In this study, using the *in vivo* imaging of heterotopically implanted CTSE-overexpressing cells in nude mice and the *in vitro* imaging of PanINs and PDAC in DMBA-induced rats, we verified the specificity of a novel synthesized CTSE-activatable probe. It was determined that this probe could identify low-grade and high-grade PanINs in addition to PDAC. In combination with CLE, we further monitored the progressive pancreatic carcinogenesis of model rats using *in vivo* imaging. With a double-blind control study and comparison with pathological data, we confirmed that CLE combined with the specific CTSE-activatable probe is a promising modality to monitor *in vivo* pancreatic carcinogenesis, and to find and identify PanINs (at low or high grade) and early PDAC. It is our hope that this approach will one day become a useful method for the regular screening of high-risk patients for PDAC.

Harshe et al. [16] carried out a systemic review of the literature, and found high expression of 162 secretory molecules and 166 membrane molecules in PDAC, some of which are highly expressed in both PDAC and PanINs, such as CTSE.

CTSE is a type of intracellular hydrolase that cleaves peptide bonds; it is not expressed in normal pancreatic tissue, but is highly expressed in PDAC and PanINs. [17]–[19] Studies have shown that CTSE expression is up to 28 times elevated in PDAC, compared to that of the normal pancreas. [20] Uno et al. [21] found that the CTSE content in pancreatic juice from patients with PDAC was significantly higher than that from patients with chronic pancreatitis. Collectively, these properties identify CTSE as a suitable biomarker for PDAC. Furthermore, we have confirmed here that the expression level of CTSE shows an increasing trend with the development of the tumor in the rat model, which suggests CTSE levels could potentially be used for the staging of PDAC and precancerous lesions in the rats.

While it is appropriate to use CTSE for cancer detection, the development of probes for CTSE-specific detection has greatly facilitated its use. However, standard fluorescence imaging is unsuitable for the *in vivo* imaging of *in situ* pancreatic tumors, due to the pancreas being located in the deep abdominal cavity. The handheld CLE probe [22]–[25] is an effective tool for *in vivo* imaging as the optical fibers can be inserted with minimal trauma. Using specific CTSE optical molecular probes with CLE, we carried out pathological prediction and comparison of pancreatic lesions based on the diagnostic classification criteria, such as the changes in fluorescence signal intensity of obtained images, cell size and morphology, and the heterogeneous pancreatic tissue structure. The diagnosis accuracy was above 82.7%, confirming the suitability of using CLE images for determining the staging of PanINs (low or high grade) and PDAC.

The use of CLE optical molecular imaging in this study was appropriate for recording the actual CTSE expression level of diseased tissue in the pancreas. While CLE has advantages, such as minimal trauma to recipients and high sensitivity, some problems with the technique still need to be resolved. These include (1) intravenous injection/local application: the local application of a molecular probe is more suitable for clinical endoscopy, as it has significantly reduced side effects when compared with systemic application [26]–[28]. However, its local application only stains the tumor surface, whereas the use of CLE allows the observation of the epithelial surface structure and the deep structure of the tumor; tomography can also be performed when compared with other optical technologies. Therefore, in this research, we injected the probes intravenously. (2) Due to the limited CLE equipment available for this study, i.e. only having one excitation wavelength of 488 nm, we had to modify our molecular probes to label it with fluorescein, which was a limitation of this study as improved penetration could have been achieved with near-infrared fluorescence imaging. This issue could be overcome by using a different instrument. (3) Although there are many research projects looking at the clinical application of CLE for human gastrointestinal diseases, it is much more complex to implement CLE imaging of the pancreas. However, this problem could be solved with the development of endoscopic techniques such as endoscopic ultrasonography-guided fine needle puncture [29]–[31], which negates the need for open surgery.

The *in situ* DMBA-induced PDAC model in rats used in this study was limited compared with the transgenic PDAC mouse model because the etiology of our model is inconsistent with that of human PDAC. But, the model used was relatively consistent with the histopathological features of human PDAC [32], [33]; PanIN grading and the formation of PDAC showed an increasing trend with the extension of DMBA induction time; and CTSE expression level was also gradually increased with the development of lesions. For the transgenic PDAC mouse model, the PDAC and PanINs often show multiple lesions and lack connective tissue hyperplasia; Some genes abnormally expressed in human PDAC are not expressed in the transgenic PDAC mouse model. [34], [35] Therefore, we performed this study using the chemical-induced model in rats on the basis of careful observation of the pathological features.

Our research has opened a new avenue for the early imaging diagnosis of PDAC, and confirmed CTSE as a biomarker for monitoring pancreatic carcinogenesis and early diagnosis of PDAC. It is our hope that an effective CLE diagnosis system for PanINs and PDAC will be established through this and more in-depth studies in the future, to facilitate the regular screening of PDAC in high-risk populations, and also the early detection and early treatment of PDAC.

## Supporting Information

Text S1 (DOCX)

Video S1 **Imaging of normal pancreatic tissue on a microscopic level.** Just a few fluorescence signals in the darkness can be visualized.
(AVI)

Video S2 **Imaging of 30th days, speculated as low grade PanINs.** CTSE-positive cells are shown.
(AVI)

Video S3 **Imaging of 60th days, speculated as high grade PanINs.** More CTSE-positive cells are shown than before and the fluorescence signal intensity is increased.
(AVI)

Video S4 **Imaging of 90th days, speculated as high grade PanINs.** More and more CTSE-positive cells are shown and the fluorescence signal intensity is further increased.
(AVI)

Video S5 **Imaging of 120th days, speculated as early PDAC.** CTSE-positive cells were diffusive, and the fluorescence signal intensity was increased and uneven.
(AVI)

Video S6 **Imaging of 150th days, speculated as PDAC.** CTSE-positive cells were strewn, and the fluorescence signal intensity was increased and uneven.
(AVI)
