# Supplementary material for: Monitoring Pancreatic Carcinogenesis by the Molecular Imaging of Cathepsin E In Vivo Using Confocal Laser Endomicroscopy
Source: PLoS One. 2014 Sep 3;9(9):e106566. doi: 10.1371/journal.pone.0106566 (PMC4153675; doi:10.1371/journal.pone.0106566)
Supplement: Text S1 — (DOCX) [file pone.0106566.s001.docx]

***In vivo* imaging DMBA-induced PDAC using CLE with the CTSE probe**

Through *in vivo* imaging of in situ DMBA-induced PDAC in a rat using CLE with the CTSE probe (probe B), continuous dynamic observation of pancreatic carcinogenesis in videos S1, S2, S3, S4, S5, S6 (CLE was carried out at various stages of pancreatic carcinogenesis at day 0, 30 days, 60 days, 90 days, 120 days, and 150 days after DMBA placement.
